# Automatic Detection of 2D and 3D Lung Nodules in Chest Spiral CT Scans

**DOI:** 10.1155/2013/517632

**Published:** 2013-02-12

**Authors:** Ayman El-Baz, Ahmed Elnakib, Mohamed Abou El-Ghar, Georgy Gimel'farb, Robert Falk, Aly Farag

**Affiliations:** ^1^Bioimaging Laboratory, Department of Bioengineering, University of Louisville, Louisville, KY 40292, USA; ^2^Urology and Nephrology Department, University of Mansoura, Mansoura 35516, Egypt; ^3^Department of Computer Science, The University of Auckland 1142, Auckland, New Zealand; ^4^Medical Imaging Division, Jewish Hospital, Louisville, KY 40202, USA; ^5^Electrical and Computer Engineering Department, University of Louisville, KY 40292, USA

## Abstract

Automatic detection of lung nodules is an important problem in computer analysis of chest radiographs. In this paper, we propose a novel algorithm for isolating lung abnormalities (nodules) from spiral chest low-dose CT (LDCT) scans. The proposed algorithm consists of three main steps. The first step isolates the lung nodules, arteries, veins, bronchi, and bronchioles from the surrounding anatomical structures. The second step detects lung nodules using deformable 3D and 2D templates describing typical geometry and gray-level distribution within the nodules of the same type. The detection combines the normalized cross-correlation template matching and a genetic optimization algorithm. The final step eliminates the false positive nodules (FPNs) using three features that robustly define the true lung nodules. Experiments with 200 CT data sets show that the proposed approach provided comparable results with respect to the experts.

## 1. Introduction

Lung cancer remains the leading cause of cancer-related deaths in the US. In 2012, there were approximately 229,447 new cases of lung cancer and 159,124 related deaths [1]. Early detection of lung tumors (visible on chest film as nodules) may increase the patient's chance of survival, but detecting nodules is a complicated task. Nodules show up as relatively low-contrast white circular objects within the lung fields. The difficulty for computer-aided detection (CADe) schemes is distinguishing true nodules from (overlapping) shadows, vessels, and ribs.

CADe systems for detection of lung nodules in thoracic CT generally consist of two major stages: (1) selection of the initial candidate nodules and then (2) elimination of the false positive nodules (FPNs) with preservation of the true positive nodules (TPNs). At the first stage, conformal nodule filtering or unsharp masking can enhance nodules and suppress other structures to separate the candidates from the background by simple thresholding or a multiple gray-level thresholding technique [2, 3]. To improve the separation, background trend is corrected in [4, 5] within image regions of interest. Then, a series of 3D cylindrical and spherical filters are used to detect small lung nodules from high-resolution CT images [6, 7]. Circular and semicircular nodule candidates can be detected by template matching [8–11]. However, these spherical, cylindrical, or circular assumptions are not adequate for describing the general geometry of the lesions. This is because their shape can be irregular due to the speculation or the attachments to the pleural surface (i.e., juxtapleural and peripheral) and vessels (i.e., vascularized) [12]. In [13, 14], morphological operators were used to detect lung nodules. The drawbacks to these approaches are the difficulties in detecting lung wall nodules. Also, there are other pattern-recognition techniques used in detection of lung nodules, such as clustering [15], linear discriminate functions [16], gray-level distance transform [17], and a patient-specific a priori model [18].

The FPNs are excluded at the second stage by nodule classification. The most popular way to do classification is to use a feature-based classifier. First, the nodule candidates identified in the first step are segmented, and features are extracted from the segmented nodule candidates. Features may include morphologic (or shape-based) features (e.g., size, circularity, and curvature), gray-level-based features (e.g., histogram-based features), and texture features. The task of the classifier is to determine “optimal” boundaries for separating classes (i.e., nodules or nonnodules) in the multidimensional feature space which is formed by the input features [19].

Recently, Dehmeshki et al. [20] proposed a shape-based template matching approach based on a genetic algorithm (GA) for the detection of spherical nodules. They compared their method, on a set of 70 CT scans, with Lee et al.'s GA template matching method [8], achieving better detection rate and lower false positives. Pu et al. [21] developed a scoring method based on the similarity distance of medial axis-like shapes obtained through a progressive clustering strategy combined with a marching cube algorithm from a sphere-based shape. Ye et al. [22] used a rule-based system followed by a weighted support vector machine (SVM) for classification. Murphy et al. [23] applied a k-nearest-neighbor classifier for classification, whereas Tan et al. [24] implemented a feature-selective classifier based on a genetic algorithm and artificial neural networks (ANNs) for classification. Messay et al. [14] developed a sequential forward selection process for selecting the optimum features for LDA and quadratic discriminant analysis. A heuristic approach was used by Riccardi et al. [25] for classification based on geometric features followed by an SVM. Thus, various approaches have been proposed for the classification component in CADe systems. However, most of these techniques do not investigate the detection of irregularly shaped nodules as well as cavity nodules.

To detect the different types of lung nodules (including small nodules, nodules attached to the wall, irregularly shaped nodules, and cavity nodules), we model nodule types with four central-symmetric deformable templates: (i) the solid spherical model of a large size (above 10 mm), calcified, and noncalcified nodules appearing in several successive slices; (ii) the hollow spherical model of large lung cavity nodules; (iii) the circular model of small nodules appearing in only a single slice; and (iv) the semicircular model of lung wall nodules. This approach allows for the isolation of abnormalities which spread over several adjacent CT slices.

Each template has a specific gray-level pattern which is analytically estimated in order to fit the available empirical data. Normalized cross-correlation is used for template matching. The 3D or 2D position, size, and gray-level pattern of each template is adjusted to the most similar part of the segmented veins, arteries, and lung abnormalities by a genetic optimization technique [26]. After all the candidates are detected, a supervised Bayesian classification of geometric and textural features of the candidate nodules partially excludes the FPNs. This paper presents an extended version of our previously published papers [27–30], containing more illustrations about each step of the proposed method as well as more experimental results to verify the accuracy and benefits of the proposed approach. 

## 2. Lung Abnormalities

In this paper, we focus on three types of lung abnormalities that can appear in spiral low-dose computed tomography (LDCT). These three types are calcified lung nodules, noncalcified lung nodules, and cavity lung nodules.

Calcification is usually detected visually when thinly collimated slices (1 to 3 mm) are performed through the nodule [31, 32]. It has recently been shown that the calcification can be inferred when a 3 to 7 mm nodule is visible on standard mediastinal images obtained using 10 mm collimation [31, 32]. Measurement of CT attenuation values can also be used to infer the presence of calcium within the nodule [31, 32]. A CT attenuation value of 200 Hounsfield units is usually used to distinguish between calcified and noncalcified nodules. Calcification of a nodule can be useful in determining benignity, although the majority of benign nodules are not calcified [31, 32]. Calcification that is diffusely solid, centrally punctuate, laminated, or “popcorn-like” in appearance is diagnostic of a benign nodule. The noncalcified lung nodules appear in the CT similar to calcified nodules but with a CT attenuation value less than 200 Hounsfield.

Cavitations occur in benign and malignant nodules and appear clearly in CT. Malignant cavities typically have thick, irregular walls, while benign cavities have smooth, thin walls [32]. For instance, 97% of cavity nodules with a wall thickness greater than 16 mm are malignant and 93% with a wall thickness less than 4 mm are benign. Cavity nodules appear in spiral CT images as hollow spheres. 

## 3. Deformable Templates of Abnormalities

**Figure 1:**
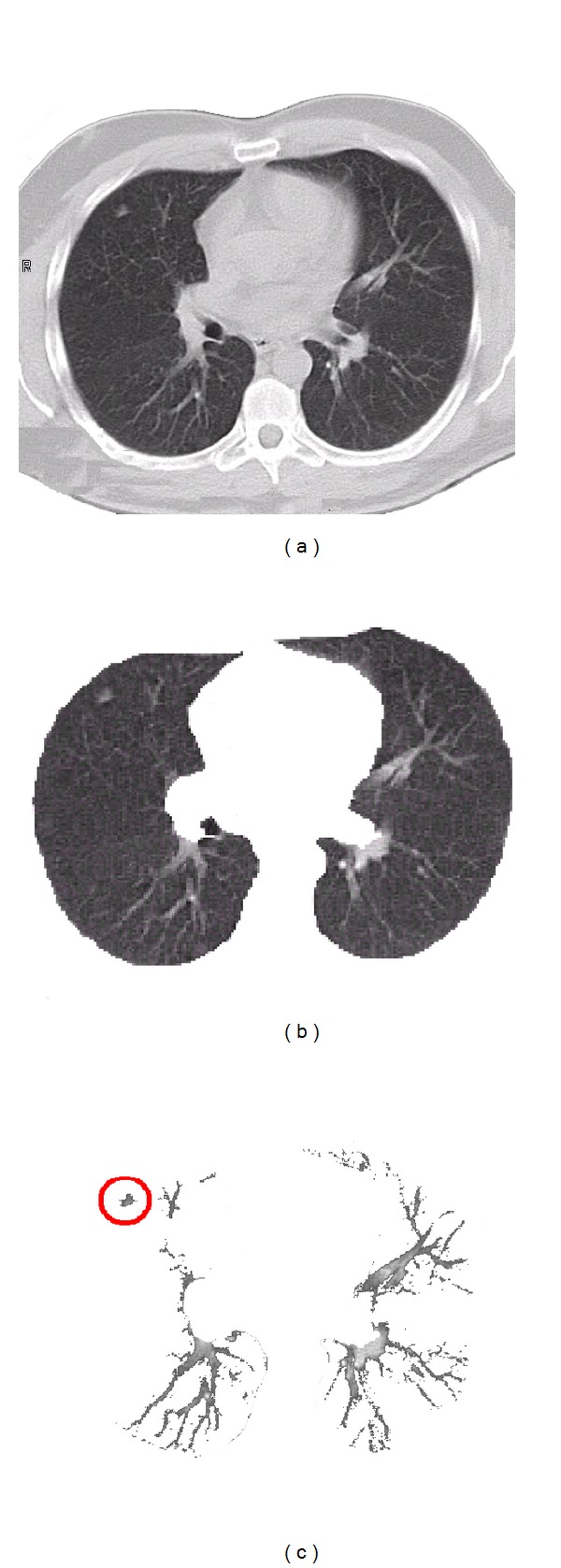
First two segmentation steps: (a) an original CT lung date sample; (b) the first segmentation step, that is, segmentation of the lung fields; and (c) the second segmentation step, that is, extracting arteries, veins, bronchi, and lung abnormalities.

 Our detection of lung nodules begins with two segmentation stages, which considerably reduce the search space. At the first stage, as shown in Figures 1(a) and 1(b), lung tissues are separated from the surrounding anatomical structures, for example, ribs, liver, and other organs, appearing in the chest LDCT scans based on an iterative Markov-Gibbs-random-field-(MGRF-) based segmentation framework, illustrated in [33]. Briefly, a linear combination of discrete Gaussians (LCDG) model [34] with positive and negative components was used to approximate the empirical distribution of the LDCT signals of the lung fields and their background, describing the 1st-order visual appearance model of the LCDG image. An initial segmentation of the lung fields was obtained by voxelwise Bayesian maximum A posteriori (MAP) classification of a given image, based on its LCDG approximation of the signals of the lung fields and their background. For accurate and smooth segmentation that retains nodules attached to the lung wall, the segmentation of the lung fields was iteratively refined by the iterative conditional mode (ICM) relaxation that maximizes a MGRF energy that accounts for the 1st-order visual appearance model and the spatial interaction between the image voxels. The second stage extracts arteries, veins, bronchi, and lung abnormalities (see Figure 1(c)) from the already segmented lung tissues based on representing each CT slice as a sample of an MGRF of region labels and gray levels. Details of the two segmentation stage algorithms are presented in [33, 34], and in this paper we focus only on the third stage of detecting and classifying the nodules among the extracted objects.

**Figure 2:**
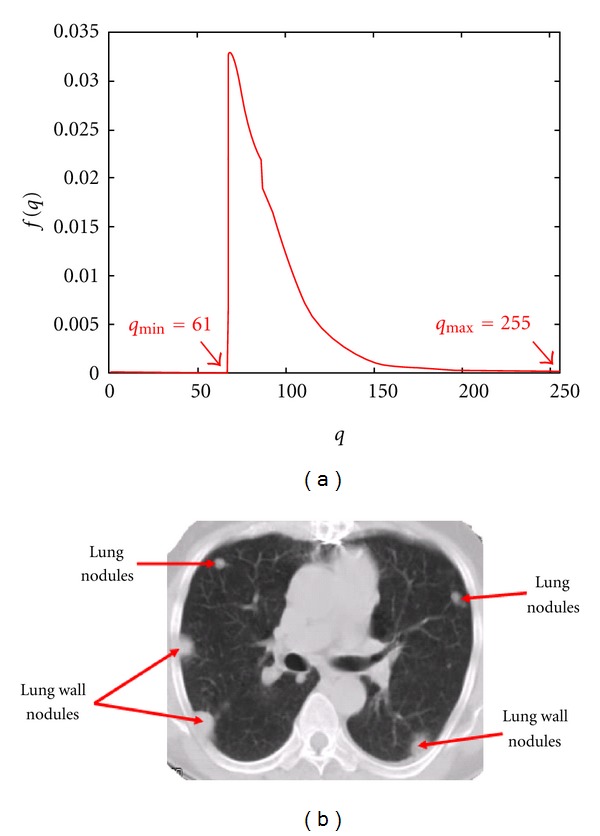
(a) The empirical gray-level distribution over the extracted regions in Figure 1(c) and (b) nodule positions and shapes.

Figure 2(a) shows the empirical gray-level distribution over the extracted regions in Figure 1(c). Both the nodules and normal tissues, such as arteries, veins, and bronchi, have almost the same gray-level distributions, so abnormality detection must include their geometry. Four basic classes of lung abnormalities are small calcified, large calcified, noncalcified, and cavity nodules. The first three classes tend to be solid spherical shapes, whereas the cavity nodules are hollow spheres.

Generally, the smaller nodules appear only in a single 2D slice like in Figure 2, whereas the larger ones spread over a 3D volume represented by several successive slices. The lung wall nodules may also appear in one or more slices, depending on their size. However, they are semicircular in shape as shown in Figure 2(b).

Our analysis of 2D CT slices suggests that spatial changes of gray-levels across the central cross-section of a solid-shape 3D nodule or across a solid-shape 2D nodule can be approximated with a central-symmetric Gaussian-like template:

(1) q(r)=qmax⁡exp⁡(−(rρ)2), 0≤r≤R.

Here, *r* is the radius from the template's center, *q*(*r*) is the gray level in a template point with Cartesian coordinates (*ξ*, *η*) with respect to the center (i.e., *r* ^2^ = *ξ* ^2^ + *η* ^2^),   *q*_max⁡_ denotes the maximum gray level for the template, *R* is the template radius depending on the minimum gray level *q*_min⁡_ = *q*(*R*), and the parameter *ρ* specifies how fast the signals decrease across the template.

## 4. Genetic Algorithm (GA) Template Matching

GA template matching is used to effectively search for the location of lung nodules that are scattered within the lung areas. In this method, the GA is used to determine the target position in an observed image and to select the proper radius to generate a template model for the template matching process. Details of the GA process are described below.

### 4.1. Template Identification

**Figure 3:**
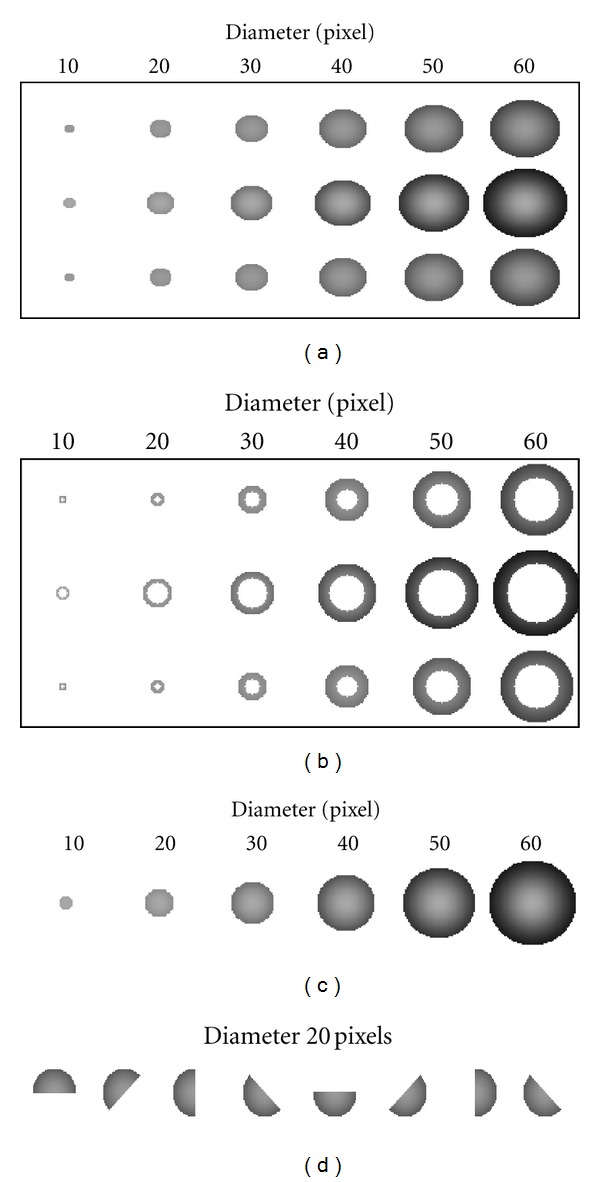
Examples of the deformed templates for GA template matching process: (a) solid spherical models consisting of three slices to detect large calcified and noncalcified nodules, (b) hollow spherical models consisting of three slices to detect thick cavity nodules, (c) circular models to detect small nodules, and (d) semicircular models to detect lung wall nodules.

The CT slices in our study have in-plane spatial resolution of 0.4 mm per pixel so that the radius range for all lung nodules is *R* = 5 − 30 pixels. The third spatial axis has lower resolution. For large solid and hollow lung nodules, we use the 3-layer template. Thin lung nodules appearing only in a single slice have the circular templates. The lung wall nodules are semicircular shapes. We assume that the template deformations, other than translations, are restricted to different scales (radii) of all the templates and also different (orientation) angles of the semicircular templates. Examples of the deformed templates are presented in Figure 3.

In order to better match between the template model and lung nodules, we have to generate templates which have densities close to the density of the segmented veins, arteries, and lung abnormalities, shown in Figure 2(a). Gray-level distribution density over the 2D Gaussian template can be found as follows:

(2) ψ(q)=2πr(q)

since $r(q)=\rho \sqrt{\ln q_{\max}-\ln q}$; then *ψ*(*q*) can be expressed as follows:

(3) ψ(q)=2πρln⁡qmax⁡−ln⁡q.

In order to compute the density for the template using (3), we need to estimate the parameter *ρ*. Following (1) for a template with radius *r* = *R*, *q*(*R*) = *q*_min⁡_ = *q*_max⁡_exp⁡(−(*R*/*ρ*)^2^), the parameter *ρ* can be estimated from the following equation:

(4) ρ=R(ln⁡qmax⁡−ln⁡qmin⁡)−1/2.

By using (4), the gray-level distribution density over the 2D Gaussian template can be expressed in the following closed form:

(5) ψ(q ∣ qmin⁡,qmax⁡)=2πRln⁡qmax⁡−ln⁡qln⁡qmax⁡−ln⁡qmin⁡.

**Figure 4:**
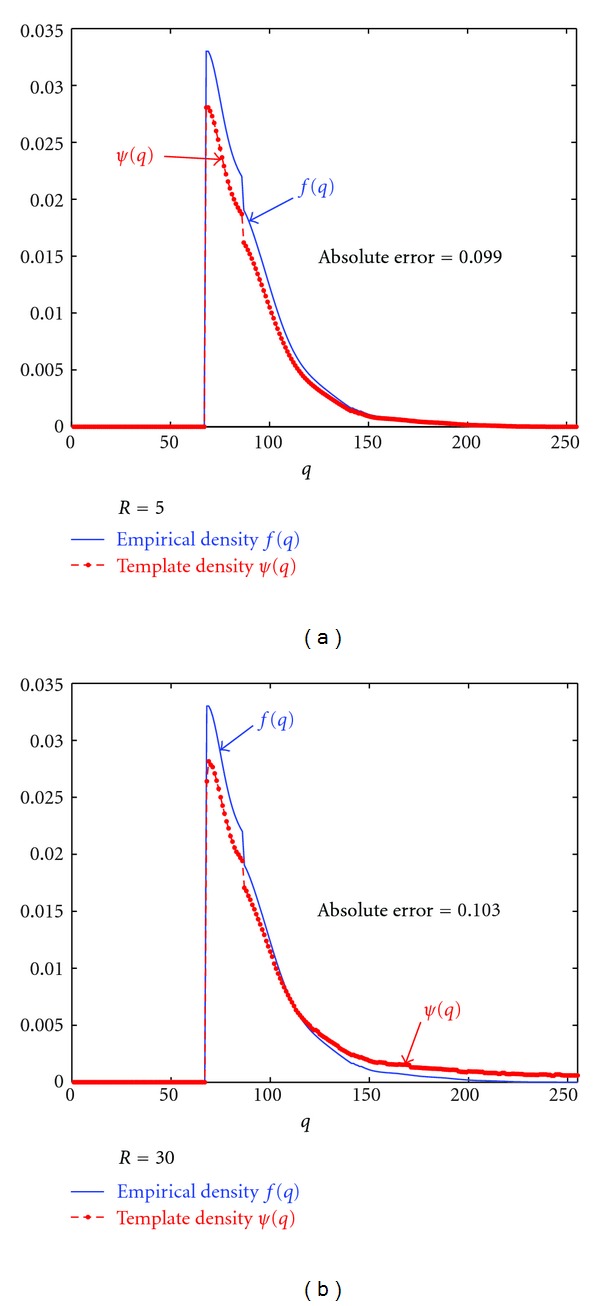
Estimated template gray-level distributions (*ψ*(*q*)) with respect to the empirical density (*f*(*q*)).

This relationship allows us to roughly estimate the template parameters *q*_max⁡_ and *q*_min⁡_ from the empirical density in Figure 2(a) (in this particular case *q*_max⁡_ = 255 and *q*_min⁡_ = 61). In particular, for the circular templates of the radii *R* = 5 and 30, the estimated *ρ* = 4.18 and 25.08, respectively.  Figure 4 shows that the estimated densities for templates using (5), with radii 5 and 30, are close to the empirical density for veins and arteries as shown in Figure 2(a). Note that the jump in the estimated density *ψ*, shown in Figure 4, is due to the fact that it is defined between *q*_min⁡_ and *q*_max⁡_.

In the case of the 3D solid spherical templates, the 2D template is first identified for the central cross-section. Next, the upper and lower cross-sections are specified by the same parameters in the following equation:

(6) qv(r)=qmax⁡exp⁡(−(r2+v2)ρ2),

where *v* is the slice thickness in pixels (*v* = 7 in our experiments below). The radius of upper and lower circles is specified by the relationship *q*_*v*_(*R*) = *q*_min⁡_.

The hollow spherical templates used to detect cavity lung nodules are obtained in a similar way by removing the central part of the solid templates up to 75% of the radius *R*.

### 4.2. The GA Template Matching Process

 As mentioned above GA is used to determine the target position in an observed image and select a suitable radius to generate a template model [28, 29]. In this paper we use the GA with the following structure (for more details about GA see [26, 35])

(i) *Chromosome*: each chromosome has 28 bits, of which 23 determine the target position and the last 5 bits determine the radius of the generated templates *R*. Furthermore, the 23 position bits are divided into 9-, 9-, and 5-bit sets corresponding to the coordinates (*x*, *y*, *z*). Once we know *R*, *q*_min⁡_, and *q*_max⁡_, we calculate *ρ* from (4). By using *ρ* and *q*_max⁡_, we generate the corresponding template. Then, similarities between the cut image (i.e., the subvolume which we cut from the original volume with the size of the generated template) and the generated template are calculated.
(ii) *Fitness*: we define the fitness of an individual as the “similarity” calculated by the normalized cross-correlation of two images, *a* and *b* [8], as
  (7) Similaritya,b=∑i=1n∑j=1n(aij−ma)(bij−mb)∑i=1n∑j=1n(aij−ma)2∑i=1n∑j=1n(bij−mb)2,

where *m*_*a*_ = (1/*n* ^2^)∑_*i*=1_^*n*^∑_*j*=1_^*n*^ *a*_*ij*_, *m*_*b*_ = (1/*n* ^2^)∑_*i*=1_^*n*^∑_*j*=1_^*n*^ *b*_*ij*_, and the values *a* and *b* signify the images for comparison. The *a*_*ij*_ is the value of pixel at site (*i*, *j*) in image *a*, similarly *b*_*ij*_. 

We particularly use GA since it is suitable for discrete optimization problems and fits the selection of candidate lung nodules, that is, select the appropriate location and radius of the lung nodule from a discrete set of candidates. The second segmentation step gives all possible candidate locations (the search space for locations). The location part in the initial population is selected randomly from these locations, whereas the radius part is selected randomly from its defined range. The next generation is formed by applying cross-over with a percentage of 75% and mutation with a percentage of 5%. If the location part in a new generated chromosome is not in the search space, we alternatively define the chromosome by its closest location on the search space. The matching algorithm runs separately for each type of lung abnormality. Note that for the semicircular template model, we add another part in the chromosome that represents the angle. All spatial locations where the similarity score is greater than a certain threshold (in our experiments 0.8) are extracted as candidate nodules.

## 5. Postclassification of Nodule Features

**Figure 5:**
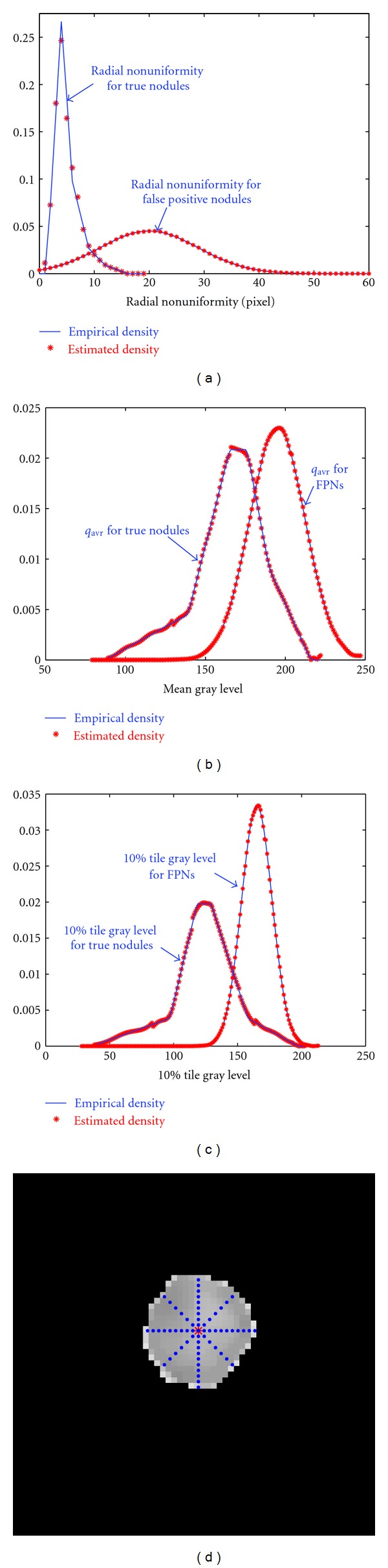
(a) Estimated and empirical density for radial nonuniformity, (b) estimated and empirical density for mean gray level, (c) estimated and empirical density for 10% tile gray level, and (d) the calculation of *d*(*θ*) at 8 directions.

**Figure 6:**
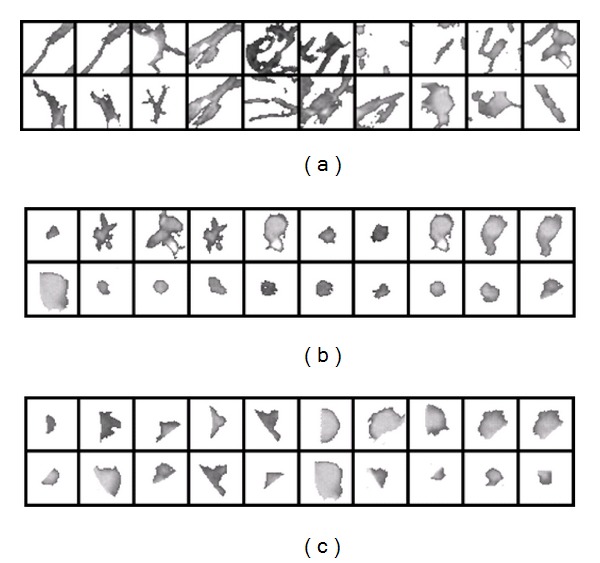
Training data to classify candidates: (a) training FPNs, (b) training lung TPNs, and (c) training lung wall TPNs.

Because actual lung nodules are not exactly spherical, circular, or semicircular, some true nodules can be missed. A number of false positive nodules (FPNs) can also be encountered during the initial extraction of the candidates. To reduce the error rate, postclassification of the candidate nodules is performed with three textural and geometric features of each detected nodule: (i) radial nonuniformity *U* = max⁡_*θ*_(*d*(*θ*)) − min⁡_*θ*_(*d*(*θ*)) of its borders; here *d*(*θ*) is the distance at the angle *θ* between the center of the template and the border of the segmented object as shown in Figure 5(d); (ii) the mean gray-level (*q*_ave_) over the 3D or 2D nodular template; and (iii) the 10% tile gray level for the marginal gray-level distribution over the 3D or 2D nodular template (a threshold value at which 10% of the nodular template points have gray-level values lower than this threshold, i.e., a threshold representing the 10% of the area under the marginal gray-level probability distribution of the nodular template). To distinguish between the FPNs and true positive nodules (TPNs), we use Bayesian supervised classifier learning statistical characteristics from a training set of false and true nodules. To train this classifier, a training set of 60 nodules was selected from 50 separate subjects, which are not included in the test. The training data are shown in Figure 6 (20 FPN, 20 lung TPN, and 20 lung wall TPN).

All three features (i)–(iii) are used to classify the FPNs in the lung, while only the last two features can be applied to the lung wall nodules. The density estimation required in the Bayes classifier is performed for each feature by using a linear combination of Gaussians (LCG) with positive and negative components. Their parameters are estimated using a modified EM algorithm which was described in [33, 34]. In this paper we assume that the three features are independent with equiprobable priors, hence the estimation for the density for each feature is done separately by using the modified EM algorithm.  Figure 5 shows the empirical and estimated densities for each feature for both TPNs and FPNs.

## 6. Experimental Results

The algorithm was tested on the CT scans of 200 subjects enrolled in the screening study. These subjects were over 60 years of age with positive smoking histories (>10 pack-years). The CT scans are collected using a screening study at the Jewish Hospital, Louisville, KY, where each patient was screened every 3 months. All nodules are validated by a radiologist (Dr. Falk, a coauthor in the paper). Small nodules (less than 3 mm) are monitored in subsequent scans, and when they reach a size of 10 mm or larger, their types are identified by a radiologist as either true nodules or not. This clinical database was collected by the LDCT scan protocol using a multidetector GE Light Speed Plus scanner (General Electric, Milwuakee, USA) with the following scanning parameters: slice thickness of 2.5 mm reconstructed every 1.5 mm, scanning pitch 1.5, pitch 1 mm, 140 KV, 100 MA, and F.O.V 36 cm. Among these 200 subjects, 21 subjects had abnormalities in their CT scans and 179 subjects were normal (this classification was validated by a radiologist). To train the second stage postprocessing classifier, another set of 50 subjects scanned with the same scanning parameters is used to train the classifier.

**Figure 7:**
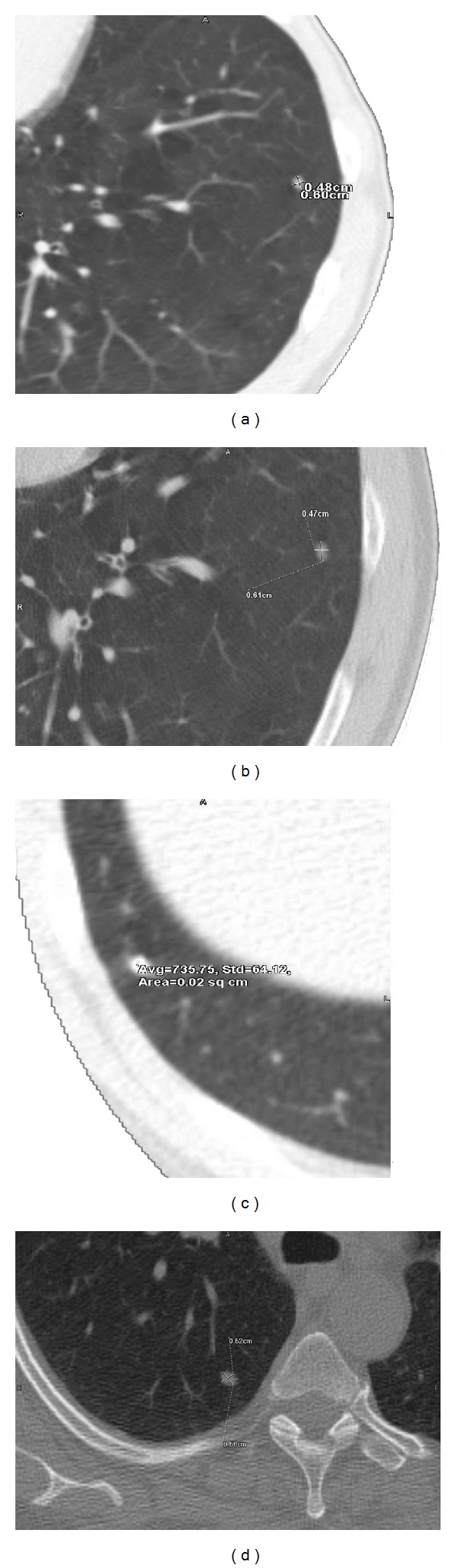
Examples nodules detected by the proposed approach. This classification was validated by a radiologist.

**Figure 8:**
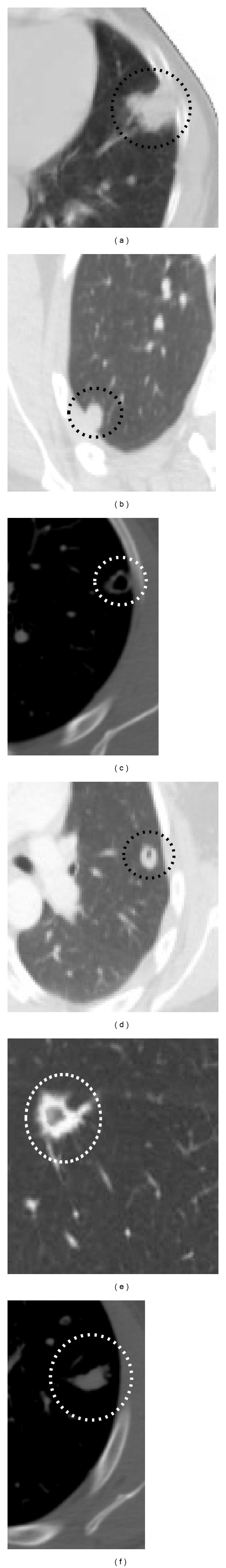
More visual results: examples of nodules attached to the wall (e.g., (a), (b), and (c)), cavitary (e.g., (c), (d), and (e)), and irregularly shaped (e.g., (e) and (f)) nodules that are successfully detected using our template matching approach. This classification was validated by a radiologist.

At stage one, the template matching extracted 110 true candidates (out of the true 130 nodules) and 49 FPNs. The classification at stage two reduced the number of FPNs to 12, but simultaneously rejected three true nodules. Thus, the final number of the TPNs became 107 out of 130, giving the overall correct detection rate of 82.3% with the FPNs rate of 9.2% (the number of FPNs with respect to the total number of true nodule, i.e., 12 out of 130). This gives a positive predictive value (PPV) of 89.9%, 107 TPNs out of a total 119 detections (107 TPNs + 12 FPNs).  Table 1 presents the numbers of TPNs and FPNs before and after the postclassification stage.  Figure 7 shows examples of small lung nodules that were detected by the proposed approach. More visual results are presented in Figure 8, where examples of cavitary nodules, irregularly shaped nodules, and nodules attached to the wall are successfully detected using our template matching approach.

To illustrate the efficiency of the proposed algorithm, we compare the results obtained by the proposed algorithm with the related work of Wang et al. [9], that detects lung nodules from a spiral CT scan using a template matching method (see Table 2). This algorithm detects only *three types* of nodules—large lung nodules, small lung nodules, and lung wall nodules—by using fixed templates. We ran Wang's algorithm on the same data sets. The algorithm detected 83 true candidates (out of the true 130 nodules) and 85 FPNs, giving the overall correct detection rate of 63.8%, a PPV of 49.4%, and a FPNs rate of 65.4%. Table 2 presents the details of the results obtained by the algorithm proposed in [9]. It is clear from Table 2 that this algorithm fails to detect large numbers of true nodules because the algorithm uses fixed-size templates in spite of employing an adaptive appearance model for the nodules. Moreover, their algorithm did not apply any postprocessing step to reduce the high rate of false positives.

## 7. Summary and Conclusion

A novel deformable template matching algorithm has been proposed for detection of lung nodules in chest CT scans. Four template shapes were used: solid sphere, hollow sphere, solid circle, and solid semicircle. The radius and the gray-scale intensity of the templates were made to vary, in order to maximize their detection capabilities. This variability in the size and shape of the templates enables detection of different types of nodules, for example, irregularly shape nodules, cavitary nodules, and small nodules (as shown in Figures 7 and 8). An analytic approach is introduced to estimate the distribution of the intensity of the templates. A preprocessing step is performed before template matching in order to isolate the lungs from the chest. Further, we isolate the homogenous tissues in the lung, which cannot be confused with the abnormalities, before template matching. The remaining lung tissues consist of blood vessels, bronchi and bronchioles, and nodules/abnormalities. This preprocessing provides data reduction of the search space before template matching and improvement in the overall detection power of the templates.

The intensity of the templates and the diameter are estimated as follows. For a given template shape (e.g., spherical), and starting from a given location (*x*, *y*, *z*) in the reduced images (CT slices), a global optimization approach is employed to choose the diameter and the intensity distribution that provides good matching (good cross-correlation with the intensity in a 64 × 64 × 3 volume centered at *x*, *y*, *z*). The location (*x*, *y*, *z*) is made to vary, and the corresponding density and diameter are estimated. Candidate templates (having the same shape, but with various diameters and intensities) are generated by the global optimization, implemented by a genetic algorithm, such that the cross-correlation is above a certain threshold (0.8 in our implementation). This step generates candidate locations of possible nodules. This process is repeated for the other template shapes. The final outcome is a number of possible/candidate lung nodules per template. A following step is implemented to reduce (ideally eliminate) all the false positive nodules (FPN), which is performed using a Bayes classifier.

Due to the nature of the search process, the speed of execution is a function of the CPU and the data size. Our present C++ implementation on the Intel dual processor (3 GHz each) with 16 GB memory and 2 TB hard drive with RAID technology takes about 5 minutes for processing 182 LDCT slices of size 512 × 512 pixels each. Current efforts are directed towards including other deformations than the radius and the intensity of the templates (e.g., jagged template shape). The results obtained have been validated by a radiologist and it is superior to what has been reported in the literature. The availability of a subject's history (e.g., chest CT scans obtained at previous times) has been shown to be an asset in improving the detection sensitivity (e.g., distinction between small nodules and bronchioles) and accuracy (e.g., reduction of false positives). We plan to incorporate this information, if available, as data fusion to our approach.

**Figure 9:**
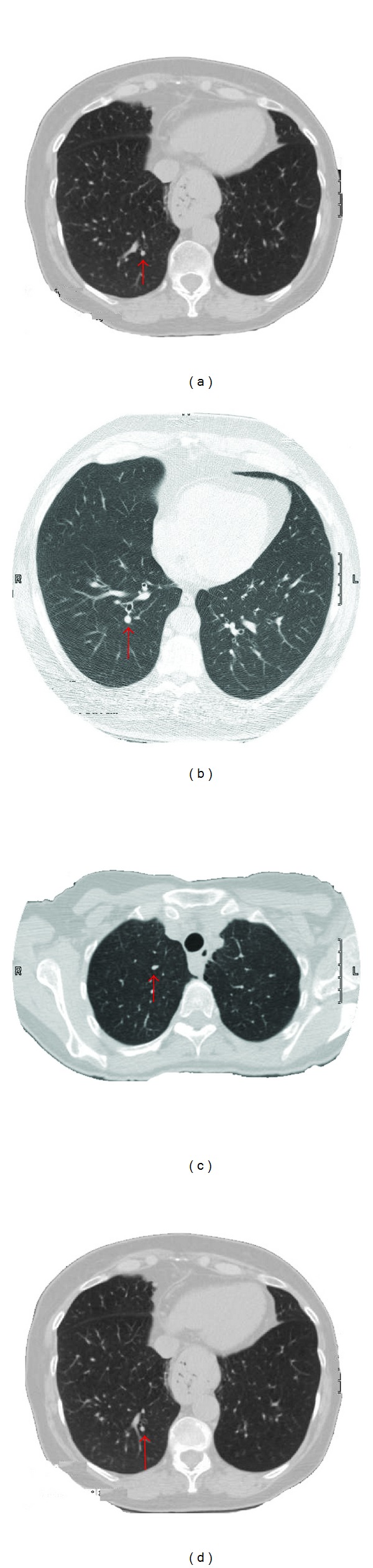
Examples of uncertainty of nodules that were not detected using our approach. The nodule's size, location, and shape resemble that of bronchi. This classification was validated by a radiologist.

Despite the large accuracy obtained by our algorithm, as compared to related work (e.g., [9]), a number of problems still persist in detecting small nodules that are confused with bronchioles as well as small blood vessels (see Figure 9, which represents examples of uncertainty of nodules that were not detected using our approach).

## Figures and Tables

**Table 1:** Detection rate for different types of abnormalities (TPNs: the nodules determined by a radiologist).

Type of lung nodules	True detecting	False detecting	True detecting	False detecting
	nodules before	nodules before	nodules after	nodules after
	removing FPNs	removing FPNs	removing FPNs	removing FPNs
Lung wall	28 : 29	8	27 : 29	2
Calcified	46 : 49	4	46 : 49	1
Non-calcified	12 : 18	5	12 : 18	3
Cavity	8 : 11	7	8 : 11	1
Small	17 : 23	25	15 : 23	5

**Table 2:** Detection rate for different types of abnormalities by using the algorithm proposed in [9] (TPNs: the nodules determined by a radiologist).

Type of lung	True detected nodules	False detected nodules
Lung wall	17 : 29	25
Calcified	39 : 49	21
Non-calcified	12 : 18	16
Cavity	—	—
Small	15 : 23	23

## References

[1] American Cancer Society (2012). *Cancer Facts and Figures*.

[2] Ko JP, Betke M (2001). Chest CT: automated nodule detection and assessment of change over time—preliminary experience. *Radiology*.

[3] Zhao B, Ginsberg MS, Lefkowitz RA, Jiang L, Cooper C, Schwartz LH Chest CT: automated nodule detection and assessment of change over timepreliminary experience.

[4] Enquobahrie AA, Reeves AP, Yankelevitz DF, Henschke CI Automated detection of pulmonary nodules from whole lung helical CT scans: performance comparison for isolated and attached nodules.

[5] Mekada Y, Kusanagi T, Hayase Y Detection of small nodules from 3D chest X-ray CT images based on shape features.

[6] Paik DS, Beaulieu CF, Rubin GD (2004). Surface normal overlap: a computer-aided detection algorithm with application to colonic polyps and lung nodules in helical CT. *IEEE Transactions on Medical Imaging*.

[7] Mendonca PRS, Bhotika R, Sirohey SA, Turner WD, Miller JV, Avila RS Model-based analysis of local shape for lesion detection in CT scans.

[8] Lee Y, Hara T, Fujita H, Itoh S, Ishigaki T (2001). Automated detection of pulmonary nodules in helical CT images based on an improved template-matching technique. *IEEE Transactions on Medical Imaging*.

[9] Wang P, DeNunzio A, Okunieff P, O’Dell WG (2007). Lung metastases detection in CT images using 3D template matching. *Medical Physics*.

[10] Ozekes S, Osman O, Ucan ON (2008). Nodule detection in a lung region that’s segmented with using genetic cellular neural networks and 3D template matching with fuzzy rule based thresholding. *Korean Journal of Radiology*.

[11] Gavrielides MA, Zeng R, Kinnard LM, Myers KJ, Petrick N A template-based approach for the analysis of lung nodules in a volumetric CT phantom study.

[12] Kostis WJ, Reeves AP, Yankelevitz DF, Henschke CI (2003). Three-dimensional segmentation and growth-rate estimation of small pulmonary nodules in helical CT images. *IEEE Transactions on Medical Imaging*.

[13] Awai K, Murao K, Ozawa A (2004). Pulmonary nodules at chest CT: effect of computer-aided diagnosis on radiologists detection performance. *Radiology*.

[14] Messay T, Hardie RC, Rogers SK (2010). A new computationally efficient CAD system for pulmonary nodule detection in CT imagery. *Medical Image Analysis*.

[15] Gurcan MN, Sahiner B, Petrick N (2002). Lung nodule detection on thoracic computed tomography images: preliminary evaluation of a computer-aided diagnosis system. *Medical Physics*.

[16] Kawata Y, Niki N, Ohmatsu H Computer-aided diagnosis of pulmonary nodules using three-dimensional thoracic CT images.

[17] Yamada N, Kubo M, Kawata Y ROI extraction of chest CT images using adaptive opening filter.

[18] Brown MS, McNitt-Gray MF, Goldin JG, Suh RD, Sayre JW, Aberle DR (2001). Patient-specific models for lung nodule detection and surveillance in CT images. *IEEE Transactions on Medical Imaging*.

[19] Duda RO, Hart PE, Stork DG (2001). *Pattern Classification*.

[20] Dehmeshki J, Ye X, Lin X, Valdivieso M, Amin H (2007). Automated detection of lung nodules in CT images using shape-based genetic algorithm. *Computerized Medical Imaging and Graphics*.

[21] Pu J, Zheng B, Leader JK, Wang XH, Gur D (2008). An automated CT based lung nodule detection scheme using geometric analysis of signed distance field. *Medical Physics*.

[22] Ye X, Lin X, Dehmeshki J, Slabaugh G, Beddoe G (2009). Shape-based computer-aided detection of lung nodules in thoracic CT images. *IEEE Transactions on Biomedical Engineering*.

[23] Murphy K, van Ginneken B, Schilham AMR, de Hoop BJ, Gietema HA, Prokop M (2009). A large-scale evaluation of automatic pulmonary nodule detection in chest CT using local image features and k-nearest-neighbour classification. *Medical Image Analysis*.

[24] Tan M, Deklerck R, Jansen B, Bister M, Cornelis J (2011). A novel computer-aided lung nodule detection system for CT images. *Medical Physics*.

[25] Riccardi A, Petkov TS, Ferri G, Masotti M, Campanini R (2011). Computer-aided detection of lung nodules via 3D fast radial transform, scale space representation, and Zernike MIP classification. *Medical Physics*.

[26] Goldberg D (1989). *Genetic Algorithms in Search, Optimization and Machine Learning*.

[27] Farag A, El-Baz A, Gimel’farb G, Falk R Detection and recognition of lung nodules in spiral CT images using deformable templates and bayesian post-classification.

[28] Farag A, El-Baz A, Gimel’Farb GG, Falk R, Hushek SG Automatic detection and recognition of lung abnormalities in helical CT images using deformable templates.

[29] Farag A, El-Baz A, Gimel'farb G Detection and recognition of lung abnormalities using deformable templates.

[30] El-Baz A, Farag A, Falk R, la Rocca R A unified approach for detection, visualization, and identification of lung abnormalities in chest spiral CT scans.

[31] Gurney JW (1993). Determining the likelihood of malignancy in solitary pulmonary nodules with Bayesian analysis. *Radiology*.

[32] Zwirewich CV, Vedal S, Miller RR, Muller NL (1991). Solitary pulmonary nodule: high-resolution CT and radiologic-pathologic correlation. *Radiology*.

[33] Farag A, El-Baz A, Gimel'farb G (2006). Precise segmentation of multi-modal images. *IEEE Transactions on Image Processing*.

[34] El-Baz A, Gimel'farb G EM based approximation of empirical distributions with linear combinations of discrete Gaussians.

[35] Michalewicz Z (1994). *Genetic Algorithm + Data Structures = Evolution Program*.

